# Ethyl 2,6-bis­(4-chloro­phen­yl)-1-iso­cyano-4-oxo­cyclo­hexa­necarboxyl­ate

**DOI:** 10.1107/S160053681401383X

**Published:** 2014-06-21

**Authors:** Dawei Zhang, Peng Yang, Wei Liu, Jing Li

**Affiliations:** a College of Agricultural Sciences, Jilin University, Changchun, Jilin Province 130062, People’s Republic of China; b College of Life Sciences and Biotechnology, Heilongjiang Bayi Agricultural University, Heilongjiang Province 163319, People’s Republic of China

**Keywords:** crystal structure

## Abstract

In the title compound, C_22_H_19_Cl_2_NO_3_, the central six-membered ring is in a twist-boat conformation. The two aryl groups are in equatorial positions, *trans* to each other and with a dihedral angle of 77.50 (2)° between them. One of the least hindered –CH_2_– groups and one of the aryl-substituted C atoms, with its axial H atom, are in the flagpole positions. The eth­oxy­carbonyl group is in an equatorial position and is *cis* to the second aryl group. In the crystal, molecules are linked *via* weak C—H⋯O hydrogen bonds, forming chains along [010].

## Related literature   

For the synthesis, see: Zhang *et al.* (2010 ▶); Tan *et al.* (2009 ▶). For related structures, see: Rowland & Gill (1988 ▶); Aleman *et al.* (2009 ▶); Wu *et al.* (2011 ▶); Li *et al.* (2011 ▶). For other [5 + 1] annulation reactions, see: Bi *et al.* (2005 ▶); Zhao *et al.* (2006 ▶); Fu *et al.* (2009 ▶); Xu *et al.* (2012 ▶).
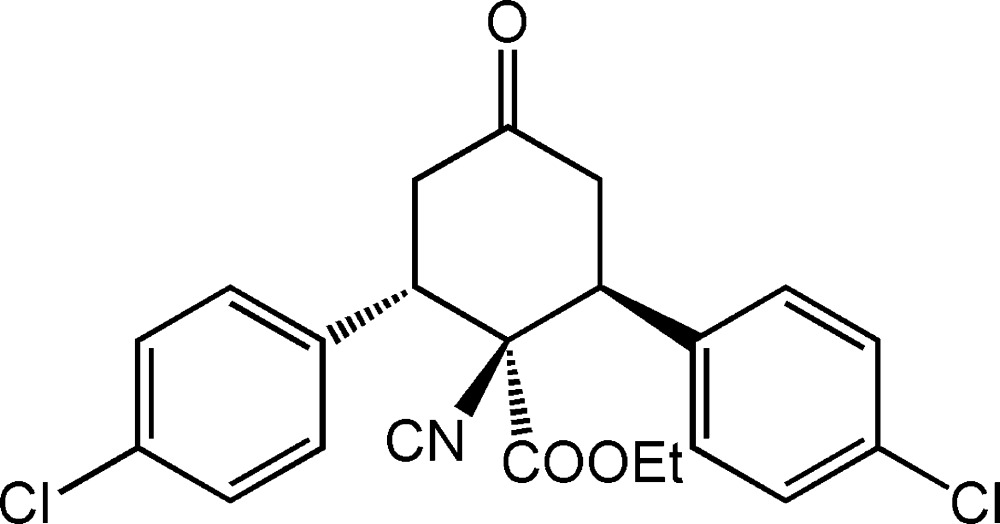


## Experimental   

### 

#### Crystal data  

C_22_H_19_Cl_2_NO_3_
*M* *_r_* = 416.28
Monoclinic, 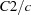

*a* = 21.6980 (17) Å
*b* = 11.0770 (19) Å
*c* = 17.515 (3) Å
β = 104.535 (2)°
*V* = 4075.0 (10) Å^3^
*Z* = 8
Mo *K*α radiation
μ = 0.34 mm^−1^
*T* = 293 K
0.21 × 0.19 × 0.15 mm

#### Data collection  

Bruker SMART APEXII CCD area-detector diffractometer
Absorption correction: multi-scan (*SADABS*; Sheldrick, 1996 ▶) *T*_min_ = 0.932, *T*_max_ = 0.951
9983 measured reflections
3602 independent reflections
2584 reflections with *I* > 2σ(*I*)
*R*_int_ = 0.027

#### Refinement  

*R*[*F* ^2^ > 2σ(*F* ^2^)] = 0.041
*wR*(*F* ^2^) = 0.117
*S* = 1.01
3602 reflections
253 parameters
H-atom parameters constrained
Δρ_max_ = 0.37 e Å^−3^
Δρ_min_ = −0.35 e Å^−3^

### 

Data collection: *APEX2* (Bruker, 2007 ▶); cell refinement: *SAINT* (Bruker, 2007 ▶); data reduction: *SAINT*; program(s) used to solve structure: *SHELXS97* (Sheldrick, 2008 ▶); program(s) used to refine structure: *SHELXL97* (Sheldrick, 2008 ▶); molecular graphics: *SHELXTL* (Sheldrick, 2008 ▶); software used to prepare material for publication: *SHELXTL*.

## Supplementary Material

Crystal structure: contains datablock(s) I. DOI: 10.1107/S160053681401383X/lr2127sup1.cif

Structure factors: contains datablock(s) I. DOI: 10.1107/S160053681401383X/lr2127Isup2.hkl

Supporting information file. DOI: 10.1107/S160053681401383X/lr2127Isup3.cml

CCDC reference: 1008201

Additional supporting information:  crystallographic information; 3D view; checkCIF report

## Figures and Tables

**Table 1:** Hydrogen-bond geometry (Å, °)

*D*—H⋯*A*	*D*—H	H⋯*A*	*D*⋯*A*	*D*—H⋯*A*
C11—H11⋯O1^i^	0.98	2.57	3.218 (3)	123

Symmetry code: (i) 
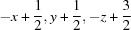
.

**Fig. 1.**
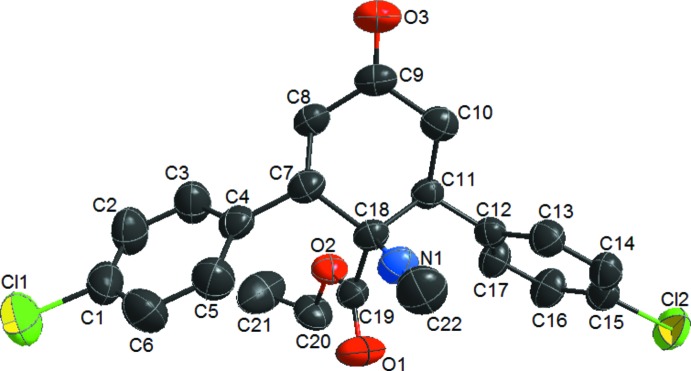
View of the molecular structure of the title compound with labeling and displacement ellipsoids drawn at the 30% probability level.

## supplementary crystallographic information

### S2.1. Synthesis and crystallization

To the mixture of 1,5-bis­(4-chloro­phenyl)­penta-1,4-dien-3-one (303 mg, 1.0 mmol) and ethyl iso­cyano­acetate (0.132 mL, 1.2 mmol) in DMF (5 mL) was added 1,8-di­aza­bicyclo [5.4.0]undec-7-ene (DBU) (0.015 mL, 0.1 mmol) in one portion at room temperature. The reaction mixture was stirred at room temperature, and the reaction mixture was monitored by TLC. After the substrate 1,5-bis­(4-chloro­phenyl)­penta-1,4-dien-3-one was consumed, the resulting mixture was poured into ice-water (30 mL) under stirring. The precipitated solid was collected by filtration, washed with water (3 × 10 mL), and dried under vacuum to afford the crude product, which was purified by flash chromatography (silica gel, petroleum ether : di­ethyl ether = 3:1, V/V) to give ethyl 2,6-bis­(4-chloro­phenyl)-1-iso­cyano-4-oxo­cyclo­hexane­carboxyl­ate (387 mg, 93%). The material was recrystallized from a mixture of petroleum ether and di­ethyl ether to provide a crystalline solid.

### S2.2. Refinement

Crystal data, data collection and structure refinement details are summarized in Table 1.Hydrogen atoms were generated in idealized positions (according to the *sp*2 or *sp*3 geometries of their parent carbon), and then refined using a riding model with fixed C—H distances (C—H = 0.95–1.00 Å) and with *U*iso(H) = 1.2*U*eq(C).

### S3. Results and discussion

[5+1] annulation is a novel strategy for the construction of six-membered cyclic compounds and total synthesis of natural products (Rowland & Gill, 1988; Wu *et al.*, 2011; Li *et al.*, 2011). The regiospecific [5+1] annulation reactions have drawn much attentions and both the five-carbon 1,5-bielectrophiles and the one-atom nucleophiles been explored extensively (Bi *et al.*, 2005; Zhao *et al.*, 2006; Fu *et al.*, 2009; Xu *et al.*, 2012). We have been dealing with functionalized ketene di­thio­acetals for several years and have succeeded in the preparation of six-membered aromatic and heterocyclic compounds based on [5C+1X] annulations (Zhang *et al.*, 2010; Tan *et al.*, 2009). The aromatic cyclic compounds are analogues of phenyl­alanine (Phe) which are potential moieties for the synthesis of peptide analogues with controlled fold in the backbone. The constrained ring systems play important roles in restricting torsional angle χ1 and in peptide receptor recognition processes (Aleman *et al.*, 2009).

The crystal structure of title compound, a phenyl substituted highly constrained cyclo­hexane analogue of Ph, is reported in this paper. Due to the steric hindrance, the oxo­cyclo­hexane is in a twist-boat conformation (Fig. 1). The ethoxyl carbonyl and the two aryl groups are located in equatorial positions. The dihedral angle between two aromatic rings is 77.495 (20)°. The C7 axial hydrogen and the CH_2_ bonded to C10 are on the flagpole positions of the boat conformation, which give the least torsional strain. The equatorial ethoxyl carbonyl on C18 and the equatorial aryl group on C11 also lead the formation of a comparable stable boat conformation of this compound.

### Crystal data

**Table:**

C_22_H_19_Cl_2_NO_3_	*F*(000) = 1728
*M**_r_* = 416.28	*D*_x_ = 1.357 Mg m^−^^3^
Monoclinic, *C*2/*c*	Mo *K*α radiation, λ = 0.71069 Å
Hall symbol: -C 2yc	Cell parameters from 106 reflections
*a* = 21.6980 (17) Å	θ = 1.3–26.0°
*b* = 11.0770 (19) Å	µ = 0.34 mm^−^^1^
*c* = 17.515 (3) Å	*T* = 293 K
β = 104.535 (2)°	BLOCK, colorless
*V* = 4075.0 (10) Å^3^	0.21 × 0.19 × 0.15 mm
*Z* = 8	

### Data collection

**Table:**

Bruker SMART APEXII CCD area-detector diffractometer	3602 independent reflections
Radiation source: fine-focus sealed tube	2584 reflections with *I* > 2σ(*I*)
Graphite monochromator	*R*_int_ = 0.027
ω scans	θ_max_ = 25.0°, θ_min_ = 1.9°
Absorption correction: multi-scan (*SADABS*; Sheldrick, 1996)	*h* = −25→25
*T*_min_ = 0.932, *T*_max_ = 0.951	*k* = −13→13
9983 measured reflections	*l* = −20→9

### Refinement

**Table:**

Refinement on *F*^2^	Primary atom site location: structure-invariant direct methods
Least-squares matrix: full	Secondary atom site location: difference Fourier map
*R*[*F*^2^ > 2σ(*F*^2^)] = 0.041	Hydrogen site location: inferred from neighbouring sites
*wR*(*F*^2^) = 0.117	H-atom parameters constrained
*S* = 1.01	*w* = 1/[σ^2^(*F*_o_^2^) + (0.0514*P*)^2^ + 2.7869*P*] where *P* = (*F*_o_^2^ + 2*F*_c_^2^)/3
3602 reflections	(Δ/σ)_max_ < 0.001
253 parameters	Δρ_max_ = 0.37 e Å^−^^3^
0 restraints	Δρ_min_ = −0.35 e Å^−^^3^

### Special details

**Table:**

Geometry. All s.u.'s (except the s.u. in the dihedral angle between two l.s. planes) are estimated using the full covariance matrix. The cell s.u.'s are taken into account individually in the estimation of s.u.'s in distances, angles and torsion angles; correlations between s.u.'s in cell parameters are only used when they are defined by crystal symmetry. An approximate (isotropic) treatment of cell s.u.'s is used for estimating s.u.'s involving l.s. planes.
Refinement. Refinement of *F*^2^ against ALL reflections. The weighted *R*-factor *wR* and goodness of fit *S* are based on *F*^2^, conventional *R*-factors *R* are based on *F*, with *F* set to zero for negative *F*^2^. The threshold expression of *F*^2^ > 2σ(*F*^2^) is used only for calculating *R*-factors(gt) *etc*. and is not relevant to the choice of reflections for refinement. *R*-factors based on *F*^2^ are statistically about twice as large as those based on *F*, and *R*- factors based on ALL data will be even larger.

### Fractional atomic coordinates and isotropic or equivalent isotropic displacement parameters (Å^2^)

**Table:**

	*x*	*y*	*z*	*U*_iso_*/*U*_eq_	
Cl2	−0.01505 (3)	0.07074 (6)	0.62309 (5)	0.0780 (2)	
Cl1	0.59500 (4)	0.06816 (9)	0.99930 (6)	0.1128 (4)	
O2	0.30900 (7)	0.21848 (13)	0.84990 (9)	0.0528 (4)	
C12	0.18929 (9)	0.19357 (17)	0.66846 (12)	0.0443 (5)	
C18	0.30971 (9)	0.15687 (17)	0.72324 (12)	0.0435 (5)	
N1	0.30407 (9)	0.04712 (16)	0.67773 (12)	0.0524 (5)	
C11	0.25594 (9)	0.24575 (18)	0.68262 (12)	0.0424 (5)	
H11	0.2578	0.3129	0.7196	0.051*	
C4	0.42949 (9)	0.17517 (19)	0.79764 (14)	0.0492 (5)	
O1	0.28944 (9)	0.02289 (15)	0.82275 (11)	0.0759 (5)	
C7	0.37764 (9)	0.21213 (18)	0.72628 (13)	0.0477 (5)	
H7	0.3901	0.1767	0.6811	0.057*	
C19	0.30148 (9)	0.12206 (19)	0.80460 (13)	0.0475 (5)	
C9	0.33050 (11)	0.3749 (2)	0.63092 (15)	0.0552 (6)	
C17	0.15361 (10)	0.2176 (2)	0.72194 (14)	0.0561 (6)	
H17	0.1719	0.2608	0.7676	0.067*	
O3	0.34259 (9)	0.45029 (17)	0.58723 (12)	0.0832 (6)	
C14	0.09922 (11)	0.0868 (2)	0.58857 (14)	0.0567 (6)	
H14	0.0811	0.0413	0.5440	0.068*	
C10	0.27117 (10)	0.2997 (2)	0.60953 (13)	0.0517 (5)	
H10A	0.2767	0.2352	0.5744	0.062*	
H10B	0.2358	0.3494	0.5818	0.062*	
C15	0.06450 (10)	0.1152 (2)	0.64196 (15)	0.0551 (6)	
C8	0.37399 (10)	0.34828 (19)	0.71039 (14)	0.0542 (6)	
H8A	0.3584	0.3888	0.7509	0.065*	
H8B	0.4162	0.3791	0.7125	0.065*	
C13	0.16142 (10)	0.12688 (19)	0.60210 (13)	0.0526 (6)	
H13	0.1849	0.1086	0.5659	0.063*	
C20	0.30188 (13)	0.2027 (3)	0.93036 (14)	0.0705 (7)	
H20A	0.2595	0.2272	0.9327	0.085*	
H20B	0.3074	0.1183	0.9452	0.085*	
C16	0.09146 (11)	0.1789 (2)	0.70886 (16)	0.0646 (7)	
H16	0.0680	0.1961	0.7453	0.078*	
C3	0.46190 (11)	0.2575 (2)	0.85150 (16)	0.0690 (7)	
H3	0.4496	0.3381	0.8459	0.083*	
C5	0.44867 (12)	0.0562 (2)	0.80921 (17)	0.0694 (7)	
H5	0.4275	−0.0025	0.7744	0.083*	
C1	0.53022 (11)	0.1084 (3)	0.92278 (17)	0.0717 (7)	
C6	0.49881 (14)	0.0224 (3)	0.87177 (19)	0.0829 (9)	
H6	0.5109	−0.0582	0.8789	0.099*	
C22	0.30053 (14)	−0.0380 (2)	0.63980 (18)	0.0765 (8)	
C2	0.51188 (12)	0.2252 (3)	0.91352 (18)	0.0793 (8)	
H2	0.5329	0.2834	0.9489	0.095*	
C21	0.34862 (18)	0.2745 (3)	0.98441 (18)	0.1104 (12)	
H21A	0.3437	0.2640	1.0370	0.166*	
H21B	0.3428	0.3581	0.9698	0.166*	
H21C	0.3905	0.2493	0.9825	0.166*	

### Atomic displacement parameters (Å^2^)

**Table:**

	*U*^11^	*U*^22^	*U*^33^	*U*^12^	*U*^13^	*U*^23^
Cl2	0.0464 (3)	0.0811 (5)	0.1010 (6)	−0.0096 (3)	0.0083 (3)	−0.0033 (4)
Cl1	0.0699 (5)	0.1466 (8)	0.1075 (7)	0.0144 (5)	−0.0045 (4)	0.0360 (6)
O2	0.0637 (9)	0.0523 (9)	0.0461 (9)	−0.0020 (7)	0.0207 (7)	0.0016 (7)
C12	0.0474 (11)	0.0384 (10)	0.0454 (12)	0.0012 (9)	0.0087 (10)	0.0009 (9)
C18	0.0492 (11)	0.0345 (10)	0.0487 (13)	−0.0035 (8)	0.0159 (10)	−0.0030 (9)
N1	0.0580 (11)	0.0386 (10)	0.0609 (12)	−0.0003 (8)	0.0153 (9)	−0.0051 (9)
C11	0.0472 (11)	0.0377 (10)	0.0428 (12)	−0.0030 (8)	0.0122 (9)	−0.0017 (9)
C4	0.0417 (11)	0.0520 (12)	0.0586 (14)	−0.0016 (9)	0.0216 (10)	0.0030 (11)
O1	0.1060 (14)	0.0511 (10)	0.0770 (13)	−0.0162 (9)	0.0350 (11)	0.0125 (9)
C7	0.0480 (12)	0.0463 (12)	0.0534 (13)	−0.0034 (9)	0.0210 (10)	−0.0009 (10)
C19	0.0430 (11)	0.0458 (12)	0.0543 (14)	−0.0024 (9)	0.0136 (10)	0.0049 (11)
C9	0.0647 (14)	0.0492 (12)	0.0582 (15)	−0.0031 (11)	0.0275 (12)	0.0072 (12)
C17	0.0511 (13)	0.0594 (14)	0.0583 (15)	−0.0059 (10)	0.0147 (11)	−0.0144 (12)
O3	0.0888 (13)	0.0857 (13)	0.0778 (13)	−0.0170 (10)	0.0257 (11)	0.0318 (11)
C14	0.0580 (14)	0.0522 (13)	0.0528 (14)	−0.0055 (10)	0.0009 (11)	−0.0060 (11)
C10	0.0611 (13)	0.0484 (12)	0.0456 (13)	0.0006 (10)	0.0133 (11)	0.0013 (10)
C15	0.0456 (12)	0.0486 (12)	0.0674 (16)	−0.0023 (10)	0.0076 (11)	0.0037 (12)
C8	0.0538 (12)	0.0498 (13)	0.0609 (15)	−0.0114 (10)	0.0179 (11)	0.0043 (11)
C13	0.0548 (13)	0.0538 (13)	0.0491 (14)	−0.0020 (10)	0.0130 (11)	−0.0039 (11)
C20	0.0799 (17)	0.0865 (19)	0.0499 (15)	0.0054 (14)	0.0254 (14)	0.0099 (14)
C16	0.0535 (13)	0.0734 (16)	0.0714 (17)	−0.0050 (12)	0.0240 (13)	−0.0131 (14)
C3	0.0583 (14)	0.0608 (15)	0.0810 (19)	0.0014 (12)	0.0046 (14)	−0.0045 (14)
C5	0.0659 (15)	0.0577 (15)	0.0817 (19)	0.0030 (12)	0.0130 (14)	0.0013 (14)
C1	0.0472 (13)	0.095 (2)	0.0732 (18)	0.0015 (13)	0.0148 (13)	0.0141 (16)
C6	0.0760 (18)	0.0686 (17)	0.102 (2)	0.0186 (15)	0.0177 (17)	0.0211 (17)
C22	0.0902 (19)	0.0528 (15)	0.083 (2)	0.0022 (14)	0.0155 (16)	−0.0109 (15)
C2	0.0608 (16)	0.087 (2)	0.080 (2)	−0.0032 (14)	−0.0005 (15)	−0.0080 (16)
C21	0.143 (3)	0.130 (3)	0.0617 (19)	−0.051 (2)	0.033 (2)	−0.021 (2)

### Geometric parameters (Å, º)

**Table:**

Cl2—C15	1.745 (2)	C14—C15	1.377 (3)
Cl1—C1	1.739 (3)	C14—C13	1.383 (3)
O2—C19	1.316 (3)	C14—H14	0.9300
O2—C20	1.466 (3)	C10—H10A	0.9700
C12—C13	1.382 (3)	C10—H10B	0.9700
C12—C17	1.383 (3)	C15—C16	1.367 (3)
C12—C11	1.519 (3)	C8—H8A	0.9700
C18—N1	1.442 (3)	C8—H8B	0.9700
C18—C19	1.529 (3)	C13—H13	0.9300
C18—C11	1.556 (3)	C20—C21	1.441 (4)
C18—C7	1.584 (3)	C20—H20A	0.9700
N1—C22	1.144 (3)	C20—H20B	0.9700
C11—C10	1.523 (3)	C16—H16	0.9300
C11—H11	0.9800	C3—C2	1.376 (4)
C4—C3	1.373 (3)	C3—H3	0.9300
C4—C5	1.382 (3)	C5—C6	1.388 (4)
C4—C7	1.513 (3)	C5—H5	0.9300
O1—C19	1.191 (3)	C1—C2	1.352 (4)
C7—C8	1.532 (3)	C1—C6	1.365 (4)
C7—H7	0.9800	C6—H6	0.9300
C9—O3	1.205 (3)	C2—H2	0.9300
C9—C10	1.500 (3)	C21—H21A	0.9600
C9—C8	1.502 (3)	C21—H21B	0.9600
C17—C16	1.378 (3)	C21—H21C	0.9600
C17—H17	0.9300		
			
C19—O2—C20	117.11 (18)	H10A—C10—H10B	108.0
C13—C12—C17	118.1 (2)	C16—C15—C14	120.7 (2)
C13—C12—C11	122.58 (19)	C16—C15—Cl2	119.88 (19)
C17—C12—C11	119.21 (19)	C14—C15—Cl2	119.39 (18)
N1—C18—C19	106.80 (16)	C9—C8—C7	110.72 (19)
N1—C18—C11	109.33 (17)	C9—C8—H8A	109.5
C19—C18—C11	109.64 (16)	C7—C8—H8A	109.5
N1—C18—C7	107.07 (16)	C9—C8—H8B	109.5
C19—C18—C7	113.03 (17)	C7—C8—H8B	109.5
C11—C18—C7	110.82 (15)	H8A—C8—H8B	108.1
C22—N1—C18	177.6 (2)	C12—C13—C14	121.2 (2)
C12—C11—C10	114.26 (17)	C12—C13—H13	119.4
C12—C11—C18	114.07 (16)	C14—C13—H13	119.4
C10—C11—C18	109.69 (16)	C21—C20—O2	109.8 (2)
C12—C11—H11	106.0	C21—C20—H20A	109.7
C10—C11—H11	106.0	O2—C20—H20A	109.7
C18—C11—H11	106.0	C21—C20—H20B	109.7
C3—C4—C5	116.7 (2)	O2—C20—H20B	109.7
C3—C4—C7	122.3 (2)	H20A—C20—H20B	108.2
C5—C4—C7	120.9 (2)	C15—C16—C17	119.6 (2)
C4—C7—C8	114.22 (18)	C15—C16—H16	120.2
C4—C7—C18	114.57 (17)	C17—C16—H16	120.2
C8—C7—C18	111.65 (16)	C4—C3—C2	122.4 (3)
C4—C7—H7	105.1	C4—C3—H3	118.8
C8—C7—H7	105.1	C2—C3—H3	118.8
C18—C7—H7	105.1	C4—C5—C6	121.4 (3)
O1—C19—O2	126.2 (2)	C4—C5—H5	119.3
O1—C19—C18	124.5 (2)	C6—C5—H5	119.3
O2—C19—C18	109.36 (17)	C2—C1—C6	120.4 (3)
O3—C9—C10	122.4 (2)	C2—C1—Cl1	119.6 (2)
O3—C9—C8	122.6 (2)	C6—C1—Cl1	120.0 (2)
C10—C9—C8	114.99 (18)	C1—C6—C5	119.5 (3)
C16—C17—C12	121.2 (2)	C1—C6—H6	120.2
C16—C17—H17	119.4	C5—C6—H6	120.2
C12—C17—H17	119.4	C1—C2—C3	119.6 (3)
C15—C14—C13	119.1 (2)	C1—C2—H2	120.2
C15—C14—H14	120.4	C3—C2—H2	120.2
C13—C14—H14	120.4	C20—C21—H21A	109.5
C9—C10—C11	111.22 (18)	C20—C21—H21B	109.5
C9—C10—H10A	109.4	H21A—C21—H21B	109.5
C11—C10—H10A	109.4	C20—C21—H21C	109.5
C9—C10—H10B	109.4	H21A—C21—H21C	109.5
C11—C10—H10B	109.4	H21B—C21—H21C	109.5
			
C19—C18—N1—C22	−164 (6)	C7—C18—C19—O2	59.8 (2)
C11—C18—N1—C22	78 (6)	C13—C12—C17—C16	−1.5 (3)
C7—C18—N1—C22	−43 (6)	C11—C12—C17—C16	175.7 (2)
C13—C12—C11—C10	41.3 (3)	O3—C9—C10—C11	−160.1 (2)
C17—C12—C11—C10	−135.7 (2)	C8—C9—C10—C11	20.6 (3)
C13—C12—C11—C18	−86.0 (2)	C12—C11—C10—C9	166.34 (18)
C17—C12—C11—C18	96.9 (2)	C18—C11—C10—C9	−64.1 (2)
N1—C18—C11—C12	55.2 (2)	C13—C14—C15—C16	−2.0 (4)
C19—C18—C11—C12	−61.5 (2)	C13—C14—C15—Cl2	177.18 (17)
C7—C18—C11—C12	173.01 (17)	O3—C9—C8—C7	−139.3 (2)
N1—C18—C11—C10	−74.4 (2)	C10—C9—C8—C7	40.1 (3)
C19—C18—C11—C10	168.84 (17)	C4—C7—C8—C9	169.06 (18)
C7—C18—C11—C10	43.4 (2)	C18—C7—C8—C9	−58.9 (2)
C3—C4—C7—C8	11.8 (3)	C17—C12—C13—C14	1.1 (3)
C5—C4—C7—C8	−164.7 (2)	C11—C12—C13—C14	−176.0 (2)
C3—C4—C7—C18	−118.7 (2)	C15—C14—C13—C12	0.7 (3)
C5—C4—C7—C18	64.7 (3)	C19—O2—C20—C21	140.9 (3)
N1—C18—C7—C4	−93.2 (2)	C14—C15—C16—C17	1.6 (4)
C19—C18—C7—C4	24.1 (2)	Cl2—C15—C16—C17	−177.61 (19)
C11—C18—C7—C4	147.63 (18)	C12—C17—C16—C15	0.2 (4)
N1—C18—C7—C8	134.96 (19)	C5—C4—C3—C2	1.1 (4)
C19—C18—C7—C8	−107.7 (2)	C7—C4—C3—C2	−175.6 (2)
C11—C18—C7—C8	15.8 (2)	C3—C4—C5—C6	−0.8 (4)
C20—O2—C19—O1	−0.1 (3)	C7—C4—C5—C6	176.0 (2)
C20—O2—C19—C18	179.17 (17)	C2—C1—C6—C5	1.3 (4)
N1—C18—C19—O1	−3.5 (3)	Cl1—C1—C6—C5	−177.6 (2)
C11—C18—C19—O1	114.8 (2)	C4—C5—C6—C1	−0.4 (4)
C7—C18—C19—O1	−121.0 (2)	C6—C1—C2—C3	−1.0 (4)
N1—C18—C19—O2	177.26 (16)	Cl1—C1—C2—C3	178.0 (2)
C11—C18—C19—O2	−64.4 (2)	C4—C3—C2—C1	−0.2 (4)

### Hydrogen-bond geometry (Å, º)

**Table:**

*D*—H···*A*	*D*—H	H···*A*	*D*···*A*	*D*—H···*A*
C11—H11···O1^i^	0.98	2.57	3.218 (3)	123

Symmetry code: (i) −*x*+1/2, *y*+1/2, −*z*+3/2.
